# On the Spikenard of the Ancients

**Published:** 1793

**Authors:** William Jones


XVI. *On the Spikenard of the Ancients. By Sir* William Jones, *Knt.* Vide *Aſiatic Reſearches: or, Tranſactions of the Society inſtituted in Bengal, for inquiring into the Hiſtory and Antiquities, the Arts, Sciences, and Literature of Aſia. Volume* II. 4to. Calcutta, 1790.

THE Indian Spikenard has long been a ſubject of uncertainty. All agree that it is an odoriferous plant, the beſt ſort of which, according to Ptolemy, grew about Rangamritica, and on the borders of the country now called Butan. It is mentioned by Dioſcorides; but his deſcription of it is very imperfect, and even Linnæus was not enabled to claſs it with certainty, though he ſuppoſed it to be a ſpecies of Andropogon. A ſimilar opinion, as we have ſeen, has lately been adopted by Dr. Biane, in an ingenious eſſay on this ſubject*;

* See vol. i, p. 153.

but but Kœnig, a diſciple of Linnæus, who died in India, aſſured the learned author of the paper now before us, that he knew not what the Greek writers meant by the Spikenard of India. Since the death of Kœnig he has conſulted, he tells us, every botaniſt and phyſician, with whom he has been acquainted, on this ſubject; but all have confeſſed, without reſerve, though not without ſome regret, that they were ignorant what was meant by the Indian Spikenard.

In order to procure information from the learned natives, it was neceſſary to know the name of the plant in ſome Aſiatic language. The very word *Nard*, the author obſerves, occurs in the Song of Solomon, but the name and the thing were both exotic. The Hebrew lexicographers imagine both to be Indian, but the word, he tells us, is in truth Perſian, and he has found it in a diſtich of an old poet, where it is not eaſy to determine whether *Nard* means the *ſtem* or the *pith*; but it is manifeſtly a part of a vegetable, and neither the *root*, the *fruit*, nor the *branch*, which are all ſeparately named. Whatever it ſignified in old Perſian, the Arabic *Sumbul*, which means an *ear* or *ſpike*, has long been ſubſtituted for it; and there can be no doubt, he thinks, that by the *Sumbul* of *India*, the Muſelmáns underſtand the ſame plant with the *Nard* of Ptolemy, and the *Nardoſtachys*, or Spikenard, of Galen, who was deceived by the dry ſpecimens he had ſeen, and miſtook them for roots.

A ſingular deſcription of the *Sumbul*, by Abu'lfazl, who frequently mentions it as an ingredient in Indian perfumes, had for ſome time almoſt convinced our author that the true Spikenard was the *Cétaca*, or *Pandanus*. His words are, *Sumbul panj berg dáred, ceh dirázii án dah angoſhteſtu pahnái ſeh*, or, "The Sumbul has five leaves, ten fingers long, and three broad." Our author was aware that the writer in queſtion, not being a botaniſt, might eaſily have miſtaken a thyrſus for a ſingle flower. He had ſeen no bloſſom, or aſſemblage of bloſſoms, of ſuch dimenſions, except the male *Cétaca*; but what moſt confirmed his opinion, was the exquiſite fragrance of the *Cétaca*-flower, which to his ſenſe far ſurpaſſed the richeſt perfumes of Europe or Aſia. Scarce a doubt remained, when he met with a deſcription of the *Cétaca* by Forſkohl, whoſe words are ſo perfectly applicable to the general idea, idea, which we are apt to form of Spikenard, that he has thought it right to give us a literal tranflation of them: "The *Pandanus* is an in-
" comparable plant, and cultivated for its
" odour, which it breathes fo richly, that one
" or two *fpikes*, in a fituation rather humid,
" would be fufficient to diffufe an odoriferous
" air for a long time through a fpacious apart-
" ment; fo that the natives in general are not
" folicitous about the living plants, but *purchafe*
" *the fpikes at a great price.*" Our author learned alfo that a fragrant effential oil was extracted from the flowers; and he procured from Banáres a large phial of it, which was adulterated with fandal; but the very adulteration convinced him, that the genuine effence muft be valuable, from the great number of thyrfi that muft be required in preparing a fmall quantity of it. Thus had he nearly perfuaded himfelf that the true Nard was to be found on the banks of the Ganges, where the Hindoo women roll up its flowers in their hair after bathing; and he imagined, that the precious alabafter box mentioned in the fcripture, and the fmall onyx, in exchange for which the poet offers to entertain his friend with a cafk of old wine, contained an effence of the fame kind with the Nard he had procured; but an Arab of Mecca, who ſaw in his ſtudy ſome flowers of the *Cétaca*, informed him that the plant was extremely common in Arabia, where it was named *Cádhi*; and ſeveral Mahomedans of rank and learning have ſince aſſured him, that the true name of the India *Sumbul*, was not *Cétaca*, but *Jatámánſi*.

Our author now abandoned his firſt opinion, and began to enquire eagerly for the Jatámánſi. A freſh plant was ſoon brought to him, and appeared, on inſpection, to be a *cyperus*, the root of which had a pungent taſte, with a faint aromatic odour; but no part of it bore the leaſt reſemblance to the drug known in Europe by the name of Spikenard; and a Muſelmán phyſician from Dehli aſſured him the plant was not *Jatámánſi*, but *Sûd*, as it is named in Arabic, which the author of the *Tohfatu'l Múmenín* particularly diſtinguiſhes from the Indian *Sumbul*: "*Sûd*, ſays that writer, has a roundiſh olive-"ſhaped root, externally black, but white in-"ternally, and ſo fragrant as to have obtained "in Perſia the name of *ſubterranean muſk*: its "leaf has ſome reſemblance to that of a leek, "but is longer and narrower, ſtrong, ſomewhat

" what rough at the edges, and tapering to a

" point. 2. *Sumbul* means a *ſpike* or *ear*, and

" was called Nard by the Greeks. There are

" three ſorts of *Sumbul*, or *Nardin*; but when

" the word ſtands alone it means the *Sumbul* of

" India, which is an herb *without flower* or

" *fruit*, (he ſpeaks of the drug only) like the

" tail of an ermine, or of a ſmall weaſel, but

" not quite ſo thick, and about the length of a

" finger. It is darkiſh, inclining to yellow, and

" very fragrant. It is brought from *Hinduſtan*,

" and its medicinal virtue laſts three years."

A ſpecimen of the dry jatámánſi was procured, and found to correſpond exactly with the above deſcription of the *Sumbul*; and our author farther learned, that the merchants brought this drug from the mountainous country to the North Eaſt of Bengal; that it was the entire plant, not a part of it, and received its Sanſcrit names from its reſemblance to *locks of hair*; as it is called Spikenard, he ſuppoſes, from its reſemblance to a ſpike, when it is dried, and not from the configuration of its flowers. The Perſian author deſcribes the whole plant as reſembling the tail of an ermine; and the Jatámánſi, which is manifeſtly the Spikenard of our druggiſts, has, it ſeems, preciſely that form, conconfifting of withered ftalks and ribs of leaves, adhering in a bundle of yellowifh brown capillary fibres, and conftituting a fpike about the fize of a fmall finger. We may, on the whole, we are told, be affured, that the *Nardus* of Ptolemy, the *Indian Sumbul* of the Perfians and Arabs, the *Jatámánſi* of the Hindus, and the Spikenard of our fhops, are one and the fame plant.

Hitherto the *Jatámánſi* has not been found growing in any part of the Britifh territories in India. Mr. Saunders, who met with it in Butan, where, as he was informed, it is very common, and whence it is brought in a dry ftate to Rangpur, fuppofed it to be a fpecies of the Baccharis ; but our author has reafon to think that the plant which Mr. Saunders faw was not the Jatámánfi, and having traced the Indian Spikenard, by this name, to the mountains of Népàl, he requefted his friend Mr. Law, who then refided at Gayá, to procure fome of the recent plants by the means of the Nepalefe pilgrims. Many young plants were accordingly fent to Gayá, where they have bloffomed. From one of thefe plants a drawing (for an engraving of which we muft refer our readers to the work itfelf,) was made by Mr. Burt, a gentleman in whofe accuracy our author expreffes the moft moſt perfect confidence, and by whoſe account he has been enabled to give a botanical deſcription of the plant. But before he proceeds to this deſcription, he endeavours to remove a prejudice, in regard to the *natural order* of the Spikenard; which they, he obſerves, who are addicted to ſwear by every word of their maſter Linnæus, will hardly abandon, and which he himſelf, he confeſſes, has abandoned with ſome difficulty. *Nard* has been generally ſuppoſed to be a *graſs*; and the word *ſtachys*, or *ſpike*, which agrees with the habit of that natural order, gave riſe, perhaps, to the ſuppoſition. There is a plant in Java, it ſeems, which moſt travellers and ſome phyſicians call *Spikenard*; and our author has been informed by the governor of Chinſura, that a Dutch author pronounces it a *graſs like the cyperus*, but inſiſts that what is called the *ſpike* is the fibrous part above the root, as long as a man's little finger, of a browniſh hue inclining to red or yellow, rather fragrant, and with a pungent, but aromatic, ſcent. From this account of it our author would have ſuppoſed, he tells us, the plant in queſtion to be the Jatámánſi, if a well-informed man, who had ſeen it in the iſland, had not aſſured him that it is a ſort of pimento, and conſequently a

ſpecies

ſpecies of myrtle. The reſemblance already mentioned between the Indian *Sumbul*, and the Arabian *Súd* or Cyperus, has led him to ſuſpect that the true Nard was a *graſs* or a *reed*; and, as Bengal abounds in odoriferous graſſes, he began to collect them from different quarters. From Colonel Kyd he received two plants with ſweet ſmelling roots; and, as they were known to the Pandits, he ſoon found their names in a Sanſcrit dictionary: one of them is called *gandháſát'hi*, and is uſed by the Hindus to ſcent the red powder of *Sapan* or *Bakkam* wood, which they ſcatter in the feſtival of the vernal ſeaſon. The other has many names, and among them *Nagaramaſtac* and *gónarda*, the ſecond of which means *ruſtling in the water*; for all the Pandits, it ſeems, inſiſt, that *Nard* is never uſed as a noun in Sanſcrit, and ſignifies, as the root of a verb, *to ſound*, or *to ruſtle*. Soon after, Mr. Burrow brought him, from the banks of the Ganges, near Heridwàr, a very fragrant graſs, which in ſome places covers whole acres, and diffuſes, when cruſhed, ſo ſtrong an odour, that a perſon, he ſays, might eaſily have ſmelt it, as Alexander is reported to have ſmelt the Nard of Gedroſia, from the back of an elephant. From Mr. Blane, of *Lucnow*, he he received a fresh specimen of the same plant. This is the species of Andropogon which Dr. Blane, as we have seen *, supposed to be the true Nardus Indica of the ancients. Lastly, Dr. Anderson, of Madras, favoured our author with a complete specimen of the *Andropogon Nardus*, one of the most common grasses on the coast, and flourishing most luxuriantly on the mountains, never eaten by cattle, but extremely grateful to bees, and containing an essential oil, which is extracted from it in many parts of Hindustan, and used as an *átar*, or perfume. He added, we are told, a very curious philological remark, that, in the *Tamul* dictionary, most words beginning with *nár* have some relation to fragrance, so that not only the *Nard* of the Hebrews and Greeks, but even the *copia narium* of Horace, may be derived from an Indian root. On this point, however, the learned author has thought it right to observe that he has not met with any such root in Sanscrit, the oldest polished language of India, and that in Persian, which has a manifest affinity with it, *nár* means a pomegranate, and *nárgil* a cocoa-nut, neither of which has any remarkable fragrance.

* Vol. i, p. 158.

None of these grasses bear any resemblance to the *Jatamansi*, which, our author conceives, to be the true Nardus of the ancients. He is not, indeed, of opinion that the Nardum of the Romans was merely the essential oil of the plant, from which it was denominated; but he is strongly inclined, he tells us, to believe, that it was a generick word, meaning what we now call *átar*, and either the *átar* of roses from Cashmir and Persia, that of Cétaca or Pandanus, from the western coast of India, or that of Aguru, or aloe-wood, from Asam, or Cochin-china, the process of obtaining which is described by Abu'lfazl, or the mixed perfume, called *ábir*, of which the principal ingredients were yellow sandal, violets, orange flowers, wood of aloes, rose water, musk, and true spikenard: all those essences and compositions, he observes, were costly; and most of them being sold by the Indians to the Persians and Arabs, from whom, in the time of Octavius, they were received by the Syrians and Romans, they must have been extremely dear at Jerusalem, and at Rome. There might also have been a pure *nardine oil*, as Athenæus calls it, but *Nardum*, our author thinks, probably meant (and Kœnig, he adds, was of the same opinion)

opinion) an Indian eſſence in general, taking its name from that ingredient, which had, or was commonly thought to have, the moſt exquiſite ſcent.

After theſe preliminary obſervations the author gives the following natural characters of the Jatamanſi.

AGGREGATE.

*Cal.* Scarce any: *margin*, hardly diſcernible.

*Cor.* One petal. *Tube* ſomewhat gibbous. *Border* five cleft.

*Stem.* Three *anthers.*

*Piſt.* *Germ* beneath. One *ſtyle* erect.

*Seed* ſolitary, crowned with a pappus.

*Root* fibrous.

*Leaves* hearted, fourfold; *radical* leaves petioled.

It appears therefore, our author obſerves, to be a ſpecies of valerian, which, in the language of Linnæus he would deſcribe, "VALERIANA JATA'MA'NSI *floribus triandris, foliis cordatis quaternis radicalibus petiolatis.*"

The radical leaves, riſing from the ground, and infolding the young ſtem, are plucked up, we we are told, with a part of the root, and being dried in the ſun, or by an artificial heat, are ſold as a drug, which from its appearance has been called *ſpikenard;* though, as the Perſian writer, already quoted, obſerves, it might be compared more properly to the *tail of an ermine;* when nothing remains but the dry fibres of the leaves, which retain their original form, they are ſaid to have ſome reſemblance to a *lock of hair*, from which the Sanſcrit name, as we have ſeen, is derived.

Two mercantile agents from Butan, on the part of the Dévárajá, or Sovereign, were examined at our author's requeſt by Mr. Harington, and informed him that the drug which the Bengaleſe call jatamanſi, "grows erect above "the ſurface of the ground, reſembling in co-"lour an ear of green wheat; that, when re-"cent, it has a faint odour, which is greatly "increaſed by the ſimple proceſs of drying it; "that it abounds on the hills, and even on "the plains, of Butan, where it is collected and "prepared for medicinal purpoſes." From Mr. Purling, who made inquiries for it among the merchants of Butan, our author had before learned that the living plant could not be obtained without an order from the Dévárajá.

